# Paediatric Burns From Deployment of a Concealed Aviation Seatbelt Airbag

**DOI:** 10.7759/cureus.15824

**Published:** 2021-06-22

**Authors:** Dujanah S Bhatti, Muhammad Adil Abbas Khan Khan, Daniel Urriza Rodriguez, Julia Cadogan, Timothy Burge

**Affiliations:** 1 Plastic Surgery, Aberdeen Royal Infirmary, Aberdeen, GBR; 2 Vascular Surgery, North Bristol NHS Trust, Bristol, GBR; 3 Paediatric Burns Service, Frenchay Hospital (North Bristol NHS Trust), Bristol, GBR; 4 Plastic and Burns Surgery, Frenchay Hospital (North Bristol NHS Trust), Bristol, GBR

**Keywords:** burn injury, air-bags, pediatric case

## Abstract

The advantages of airbags in reducing the rate of severe injuries and fatalities in motor vehicle crashes are well known but the physical act of airbag deployment can lead to injury to the passenger and the spectrum of airbag trauma resulting from deployment of vehicle airbags has been extensively reported. We present the first reported case of a pediatric burn injury resulting from the accidental deployment of an airbag in an aircraft. A four-year-old female child sustained injuries to the left side of her face and body while she was aboard a stationary airplane and the airbag concealed within the seat belt of her airplane seat unexpectedly and inexplicably deployed just before departure. We are presenting the case to increase awareness of the possibility of this injury in aircraft and to enable minimization of such accidents as well as help establish protocols for dealing with such mishaps if there were to happen.

## Introduction

Since their introduction airbags have become common and have led to a considerable decline in trauma-related injuries and deaths in motor vehicle accidents. Despite their usefulness, airbag deployment can lead to a number of potential injuries which includes trauma resulting from the explosion at deployment, the hot gases generated during inflation of the airbag, the corrosive alkaline chemical plume created as a combustion by-product, and also the blunt mechanical trauma from the sudden expansion of the airbag itself. The severity of passenger injury depends on the rate of an airbag deployment, chemicals used within the device, and the design of the device and seat belt.

The spectrum of resultant injury can include cutaneous burns due to a combination of thermal, chemical, and mechanical (friction-abrasion type) aetiologies and airway injury from thermal and chemical insult. A number of other injuries including ocular and auricular trauma, facial, long bone or rib fractures, and internal organ injury from blunt trauma are possible and warrant a high level of suspicion, immaculate clinical and radiologic assessment, and subsequent management of these cases.

Airbag injuries resulting from motor vehicle trauma have been extensively reported in medical literature but there has been no formal report of injuries resulting from airbag deployment in an aircraft. Here, we present the first case of trauma to a pediatric patient resulting from the accidental deployment of an airbag on an aircraft and summarise a review of relevant literature. Our aim is to highlight the possibility of this injury to help airlines develop safety measures and protocols to deal with these injuries if they were to happen and also to enable airbag manufacturers to develop the technology of aviation airbags to prevent accidental deployment of airbags on aircraft.

## Case presentation

We here describe the case of a four-year-old female child who suffered burn injuries while onboard a stationary aircraft on her way home after a holiday abroad. The airbag was concealed within her seat belt unexpectedly and inexplicably deployed before departure, resulting in burn injuries to the left side of the face, left jawline, left side of the chest, left upper limb, and left anterior thigh. Before departure, she was assessed by paramedics and deemed fit to undertake the eight-hour transatlantic flight to the United Kingdom.

On landing, she was referred to the local hospital emergency department where she was seen approximately 12 hours after the injury. She was in pain but was hemodynamically stable and had a patent airway. She was noted to have cutaneous injuries with associated swelling on the left side of her face and body but there was no obvious auditory, ophthalmic, dental, or internal organ trauma on clinical examination and no associated fractures on radiologic assessment. Her past medical history was unremarkable, with no regular medication or known drug allergies and she had normal developmental milestones. She was referred to a specialist center for a review of her cutaneous injuries, given oral morphine for her pain, and transferred across by ambulance.

**Figure 1:**
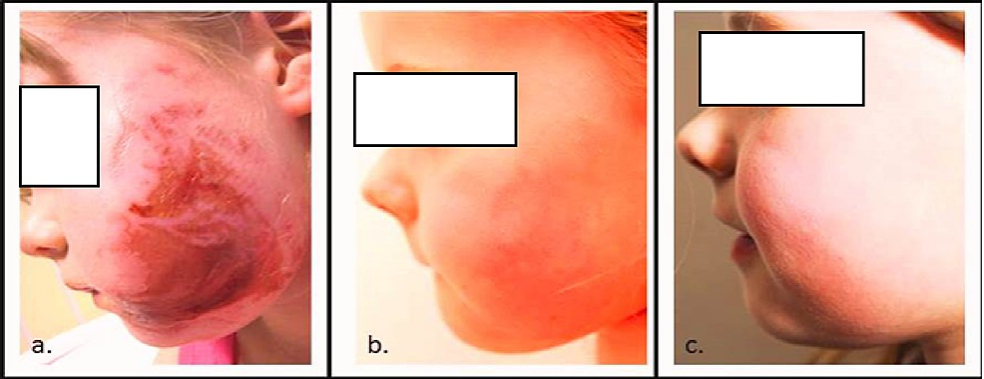
Progression and stages of healing. (a) Two days after injury; (b) two months after injury; (c) one year after injury.

Detailed examination of her cutaneous injuries at the specialist center revealed a combination of blistering, friction-abrasion type injuries with bruising and swelling on the left malar area, cheek, and chin which were consistent with superficial partial-thickness burns. There was a strip of skin overlying the left jawline where the burn was slightly deeper and was clinically consistent with a partial thickness burn (Figure 1a). There was a small zone of contusion without epidermolysis over the left upper chest consistent with blunt trauma. Patchy non-circumferential superficial partial-thickness burns were also noted on the anterior and medial aspect of the left upper limb extending in a patchy distribution from the anterior left axilla down to the left wrist along the medial border of the limb. There were no associated fractures but the range of motion was limited at the left elbow and wrist secondary to pain. A patch of the friction-abrasion-type burn was also seen over her left anterior thigh. There were no concerns regarding inhalational injuries on clinical examination and a formal airway assessment was not warranted. The pH of the burn sites was normal but suspecting a combination etiology (thermal, chemical, and mechanical burns), the burn sites were cleaned with saline and the facial burns were treated with Vaseline and non-adherent silicone dressings were applied to the patchy burn on her left upper limb. The left upper limb was placed in a sling for comfort and she was admitted to the hospital for analgesia, observation, and to facilitate feeding. She also underwent an assessment by a pediatric clinical psychologist to help her and her parents deal with the shock of her accident and injuries. Her inpatient stay was unremarkable, there were no safeguarding concerns and the child was discharged after 48 hours later. She remained well and her occasional complaints of itching at the burn sites were responsive to oral antihistamines.

A week after the injury at the first out-patient clinic review, the burns sites appeared to be healing with no evidence of infection. The non-adherent dressings applied to the sites on her left upper limb were replaced and the parents were advised to continue applying Vaseline to the face. The child had had some difficulty eating and drinking and the parents had encouraged oral intake through a straw which she had managed. Sleep disturbance, enuresis, clinginess, and fear of heat were reported on review by the pediatric clinical psychologist. Two weeks after the injury, at the second out-patient review, the burn sites had healed (Figure 1b). The child was referred to the scar management team who advised the use of moisturizer over the zone of injury and recommended sun protection.

Five weeks after the injury, a third out-patient clinic review was undertaken as the child’s brought forward concerns regarding the “glowing red” appearance of the healed areas on the left side of the child’s face and jawline when the child was physically active or was flustered. On examination, no hypertrophy of the healed areas was observed. The parents were reassured that the scar was in its remodeling phase and may appear red in warm temperatures or appear dusky in cold weather for up to 12-24 months after the injury (Figure 1c). The need for regular application of a non-perfumed moisturizer and the importance of sun protection was reinforced. On subsequent visits, the complaint was still there, flushing in warm weather and mild mottling (“blemish”) in cold weather. The senior author referred to this phenomenon as “intermittent dermal hypervascularity” and felt that it would settle in 18-24 months, which it did.

The child remained fearful of aircraft despite having traveled on them after the injury. She also suffered nightmares for months and slept in with her mum and dad for the first eight months and after that preferred to sleep with her younger brother. Her parents explained that she had been so severely emotionally affected by the accident that she continued to talk about the incident daily for a few years after the accident. In this regard, the early involvement and follow-up by a pediatric clinical psychologist were greatly beneficial to the child’s recovery.

## Discussion

Airbags were introduced in 1953 [1] and a subsequent increase in their use has been observed. Multiple countries have made regulations for all automobiles to be fitted with airbags as they significantly reduce the impact of an accident [2,3] resulting in a decline in trauma-related injuries and deaths [1,4]. The installation of airbags in aircraft is comparatively new following the Federal Aviation Administration, USA prescription that all airplane seats must withstand impacts and stress of up to 16 times the force of gravity as this reduces the force on a person in the case of an accident, leading to less severe internal injuries and increasing the chance of survival. Some airlines now have incorporated airbags into their airplane seatbelts to meet this requirement. The device contains sensors that can detect an impending accident and deploy up and away from the seated passenger to enable protection to the upper body of the passenger. However, in the case of our patient, the seatbelt was faulty and the airbag was deployed without a trigger.

Medical literature relevant to airbag injuries is exclusive to automobile airbags and has shown that airbags, when used as an adjunct to seatbelts, considerably reduce the incidence of fatal injuries when compared to seatbelts alone in automobile accidents [5-7]. There has been no formal reporting in the medical literature of airbag-related injuries in aircraft to date and the evidence quoted in this manuscript pertains exclusively to airbag design and automobile accidents. Children may be at significant risk of injury from airbag deployment and recommendations are that children should ride in the back seat of vehicles, restrained by seat belts, and away from airbags.

Airbags inflate when a collision sensor activates a detonator which causes an explosion of sodium azide resulting in the production of multiple gases [1,7,8] including nitrogen, carbon monoxide, carbon dioxide, nitric oxide, etc. These hot gases inflate the airbag at velocities of up to 200-300 km/hour [9] and temperatures as high as 500-4000 °C are achieved [7,8]. The hot gases can result in thermal injury and the explosion can ignite gasoline fumes or other flammable components of the vehicle, placing passengers at risk for serious burns. After inflation, these gases are released from vents in the airbag within the subsequent two seconds [8,10] to produce space for the passenger. The airbag material is coated with talcum powder to enable its release from its container and when the airbag deploys with great force, the powder creates a dust cloud which can add a chemical injury component on top of the thermal injury. Nylon, melting point 265 °C, is the fabric used to manufacture these airbags [2,3], and there is a chance of a tear due to high pressures generated leading to the release of the alkaline substances such as sodium azide or sodium hydroxide from it [2]. The hot airbag material can also result in friction abrasion injuries to the passenger. The inflation force of airbag deployment can reach speeds of up to 200 mph and this can result in fractures or internal injuries as a result.

The mechanism of airbag-associated injuries suggests that they are not due to malfunctioning but rather due to their intrinsic design [8]. The nature and extent of these injuries depend greatly on the posture and size of the passenger, the rate of an airbag deployment, chemicals used within the device, and the design of the device and seat belt [11].

The distribution of traumatic injuries mostly involves the face, chest, and upper extremities [12,13], and the spectrum of reported injuries include abrasions, lacerations, dental, auricular and ocular trauma, blunt trauma internal organ injuries including pulmonary contusions, arterial dissection, pericardio-diaphragmatic rupture, laryngotracheal disruption, and skeletal injuries including fractures of the temporomandibular joint, sternum ribs and upper extremity [12-15]; 7.8% of airbag injuries result in burns (7.8%) [3,7].

The burn injuries [2,7,12,14] resulting from airbag deployment have been classified by Hallock into three main categories; thermal, chemical, and mechanical (friction-abrasion) burns [10,13]. These burns range from superficial partial-thickness burns [3,12] to full-thickness burns. Like other injuries, burn injuries are seen more frequently on the face, chest, upper extremities, and abdomen [8,15] as was the case in our patient.

The temperatures generated during inflation of the airbags can be as high as 4000 °C and this can lead to thermal injuries [3,7-8]. Thermal injuries can be due to (a) direct contact burn from the hot material of the airbag as the maximum temperature of the surface of an airbag can be as high as 92 ± 2 °C during inflation [16]; (b) exposure to the hot gases generated for inflation of the airbag; (c) melting of passenger clothing, e.g., polyester due to thermal exposure [10]. Chemical burns can be attributed to the release of corrosive alkaline substances [1] such as sodium azide from the bag. Water may react with sodium azide to form highly toxic hydrazoic acid which causes more injury. Chemical injuries are more common in cases where the airbag ruptures leading to the release of these substances [1] and superficial partial-thickness and mixed depth burns resulting from chemical exposure have been reported [8]. In cases where chemical injuries are suspected, the use of copious irrigation has been advised. Mechanical or friction-abrasion injuries result from the large number of forces produced by the expanding airbag. The expansion speed of 200-300 km/hour can exert mechanical forces on contact with the passenger resulting in friction-abrasion injuries. Most of these burns require conservative management and heal quickly [12] without leaving scars, however, in some cases scars are persistent [14,17,18].

In addition to the physical impact of these injuries, there are also likely psychological issues in the short, medium, and long term. These are an important concern in pediatric patients and commonly include intrusive memories of the event; sleep disturbance, behavioral changes, and raised levels of anxiety, particularly if exposed to direct or indirect reminders of the mechanism of the injuries and treatment [19]. When these symptoms persist longer than several weeks, a diagnosis of post-traumatic stress disorder may be warranted [20] and it becomes important for psychological support to be provided to the child and family to reduce the risk of recurring and chronic symptoms which may, in some instances, severely affect a child’s confidence and ability to cope with situations which evoke memories of past events.

Our patient had no residual scars from the burn injuries and the psychological issues were addressed by an early referral to a pediatric clinical psychologist who followed the patient and reported a positive outcome on long-term follow-up.

## Conclusions

Our case is the first reported case of pediatric burn injuries resulting from the accidental deployment of an airbag in an aircraft. Though airbags are a great improvement to automobiles there remains a need for improvement in the design of the airbags. We hope that with the awareness we bring forward with this paper, improvements will be made to airbag design by manufacturers to make them safer and prevent such accidents from happening again. We are also hopeful that formal documentation of this entity in a medical journal shall enable airlines to recognize this potential injury as a possibility and put into place safety measures and protocols in place to deal with these injuries if they were to happen on board.

## References

[1] Baruchin AM, Jakim I, Rosenberg L, Nahlieli O (1999). On burn injuries related to airbag deployment. Burns.

[2] Masaki F (2005). A new category of contact burn resulting from air bag infusion. Burns.

[3] Tsuneyuki Y, Gozo N, Masaki F, Osamu M (2003). Facial contact burn caused by air bag deployment. Burns.

[4] Wallis LA, Greaves I (2002). Injuries associated with airbag deployment. Emerg Med J.

[5] Zador PL, Ciccone MA (1993). Automobile driver fatalities in frontal impacts: air bags compared with manual belts. Am J Public Health.

[6] Cummins JS, Koval KJ, Cantu RV, Spratt KF (2011). Do seat belts and air bags reduce mortality and injury severity after car accidents?. Am J Orthop (Belle Mead NJ).

[7] Mercer GN, Sidhu HS (2005). Modeling thermal burns due to airbag deployment. Burns.

[8] Sinha VK, MacGill KA (2007). Air bag-associated burn. Emerg Med Australas.

[9] Agusti-Mejiasa A, Messeguerb F, García-Ruiza R, de la Cuadraa J, Pérez Ferriolsa A, Alegre-de Miquel V (2010). [Chemical burn from an airbag]. Actas Dermosifiliogr.

[10] Hallock GG (1997). Mechanisms of burn injury secondary to airbag deployment. Ann Plast Surg.

[11] Hault-Dubrulle A, Robache F, Delille R (2012). Influence of pre-crash driver posture on injury outcome: airbag interaction with human upper extremities. Comput Methods Biomech Biomed Engin.

[12] Ulrich D, Noah EM, Fuchs P, Pallua N (2001). Burn injuries caused by air bag deployment. Burns.

[13] Corazza M, Trincone S, Zampino MR, Virgili A (2004). Air bags and the skin. Skinmed.

[14] Suhr M, Kreusch T (2004). Burn injuries resulting from (accidental) airbag inflation. J Craniomaxillofac Surg.

[15] Swanson-Biearman B, Mrvos R, Dean BS, Krenzelok EP (1993). Air bags: lifesaving with toxic potential?. Am J Emerg Med.

[16] Shakouri E, Mobini A (2019). Airbag deployment: infrared thermography and evaluation of thermal damage. Proc Inst Mech Eng H.

[17] Auh E, Kistamgari S, Yang J, Smith GA (2020). Children with facial burns treated in united states emergency departments, 2000 to 2018. Acad Pediatr.

[18] Wang H, Song G, Ren W (2018). Traumatic facial fractures in children and adolescents. J Craniofac Surg.

[19] Bakker A, Maertens KJ, Van Son MJ, Van Loey NE (2013). Psychological consequences of pediatric burns from a child and family perspective: a review of the empirical literature. Clin Psychol Rev.

[20] Stoddard FJ Jr, Sorrentino E, Drake JE (2017). Posttraumatic stress disorder diagnosis in young children with burns. J Burn Care Res.

